# Rubella immunity among prenatal women in Ontario, 2006–2010

**DOI:** 10.1186/1471-2334-13-362

**Published:** 2013-08-02

**Authors:** Gillian H Lim, Tara Harris, Shalini Desai, Natasha S Crowcroft, Tony Mazzulli, Tina Kozlowski, Shelley L Deeks

**Affiliations:** 1 Immunization and Vaccine Preventable Diseases, Public Health Ontario, 480 University Avenue, Suite 300, Toronto M5G 1V2, Canada; 2 Centre for Immunization and Respiratory Infectious Diseases, Public Health Agency of Canada, 180 Queen Street West, 11th floor, Toronto M5V 3L7, Ontario, Canada; 3 Infectious Diseases, Public Health Ontario, 480 University Avenue, Suite 300, Toronto M5G 1V2, Canada; 4 Dalla Lana School of Public Health, University of Toronto, 155 College Street, Toronto M5T 3M7, Canada; 5 Department of Laboratory Medicine and Pathobiology, University of Toronto, 1 King’s College Circle, 6th Floor, Toronto M5S 1A8, Canada; 6 Public Health Ontario Laboratory, 81 Resources Road, Etobicoke M9P 3T1, Canada; 7 Department of Microbiology, Mount Sinai, 600 University Avenue, Room 210, Toronto M5G 1X5, Canada; 8 Department of Medicine, University of Toronto, 200 Elizabeth Street, Suite RFE 3-805, Toronto M5G 2C4, Canada

**Keywords:** Rubella, Seroprevalence study, Prenatal screening, Rubella elimination goals, Ontario, Canada

## Abstract

**Background:**

Countries of the Americas have been working towards rubella elimination since 2003 and endemic rubella virus transmission appears to have been interrupted since 2009. To contribute towards monitoring of rubella elimination, we assessed rubella seroprevalence among prenatal screening tests performed in Ontario.

**Methods:**

Specimens received for prenatal rubella serologic testing at the Public Health Ontario Laboratory, the provincial reference laboratory, between 2006 and 2010 were analyzed. A patient-based dataset was created using all tests occurring among 15–49 year-old females, where prenatal screening was indicated. Multiple tests were assigned to the same patient on the basis of health card number, name and date of birth. Only unique tests performed at least nine months apart were included. SAS version 9.2 was used for analysis.

**Results:**

Between 2006 and 2010, we identified 459,963 women who underwent 551,160 unique prenatal screening tests for rubella. Of these, 81.6%, 17.1% and 1.4% had one, two and three or more tests respectively.

Rubella immunity remained stable at approximately 90% overall; the proportion of susceptible women was 4.4%. Additionally, 0.6% of women were initially susceptible and subsequently developed immunity. Across the province, susceptibility was highest in the north and declined with increasing age (p < 0.0001). Among women with multiple tests, the proportion who remained susceptible declined as the number of years between tests increased (p < .0001). Based on age at first test, younger women had the highest susceptibility (4.2% among 15–19 year-olds) and were significantly more likely to develop immunity if previously susceptible (p < .0001).

**Conclusion:**

Rubella susceptibility among prenatal women in Ontario supports elimination goals as population immunity in this group is relatively high. Higher susceptibility among young women and women living in the north highlights an opportunity for greater focus on identification and immunization of susceptible women in these groups.

## Background

Countries of the Americas have been working towards the goal of eliminating rubella and congenital rubella syndrome (CRS) since 2003 and endemic rubella virus transmission appears to have been interrupted since 2009 [1,2]. With the guidance of a Plan of Action from the Pan American Health Organization (PAHO), member states are currently documenting and verifying interruption of endemic rubella virus transmission in their respective jurisdictions [2]. The Plan of Action describes six components which will provide support that measles and/or rubella/CRS has been eliminated. This includes high population immunity demonstrated by immunization coverage estimates and supported by seroprevalence studies where available.

In Canada, rubella immunization coverage goals were set in 2005 to achieve and maintain 97% coverage for one dose of rubella-containing vaccine among children by their second birthday, and 97% coverage for two doses of rubella-containing vaccine among 7 and 17-year olds by 2010 [3]. In Ontario, Canada’s largest province (population 13.7 million), rubella-containing vaccine has been administered as part of publicly funded immunization programs since 1970. Introduced in 1975, it has been administered as part of a one-dose schedule of the combined measles, mumps, rubella (MMR) vaccine. To improve measles control, a two-dose MMR program was introduced in 1996, where the first dose was administered at 12 months and the second dose was administered at 4–6 years until 2007, where the second dose was administered at 18 months. As of August 2011, the second dose is administered as a combined measles, mumps, rubella and varicella (MMRV) vaccine among 4–6 year olds. A single dose of monovalent measles vaccine was offered to all students aged 4–18 years in 1996 as part of a measles catch-up campaign. In Ontario, as legislated by the Immunization of School Pupils Act, immunization with at least one dose of rubella-containing vaccine is required for school attendance, unless a valid medical exemption or statement of religious or conscientious objection is provided.

One imported case of congenital rubella syndrome and 12 confirmed cases of rubella were reported in Ontario between 2006 and 2011; none of these cases were determined to be endemic [4]. The last reported case of rubella in Ontario occurred in January 2012 and was assessed to be travel-related [5]. Immunization coverage for at least one dose of rubella-containing vaccine was estimated to be 95.0% and 96.6% among children 7 and 17 years of age respectively during the 2010–11 school year [6]. Unfortunately, as Ontario does not have a comprehensive immunization registry, coverage among pre-school children or adults cannot be assessed. At the national level, self-reported data obtained through telephone surveys estimate one-dose coverage of MMR vaccine as 92% among 2 year olds in 2009 [7] and 71% among adults < 38 years in 2008 (personal communication, S. Desai). National targets have been set to decrease susceptibility among primigravida women to less than 4% and to achieve 99% coverage in susceptible women postpartum [3].

Seroprevalence can provide additional evidence of population immunity particularly among specific target groups for immunization; because of primary and secondary vaccine failure, coverage only provides a proxy for immunity. In Canada, it is recommended that all pregnant women are screened to determine susceptibility to rubella and facilitate post-partum immunization of susceptible women, increasing the feasibility of assessing rubella seroprevalence [8,9]. A small number of Canadian studies have assessed seroprevalence of rubella in selected adult populations including military recruits, daycare workers and newly arrived immigrants and refugees [10-12] but only a few studies have specifically assessed pregnant women [13-15].

The objectives of our study are to determine rubella susceptibility in a sample of prenatal rubella screening tests conducted in Ontario; to identify demographic factors associated with non-immune rubella titres and contribute towards monitoring of rubella elimination in Ontario and Canada.

## Methods

In Ontario, prenatal specimens as well as those for occupational health pre-employment screening, are sent to the provincial reference laboratory, Public Health Ontario Laboratory (PHOL) for serologic testing. We analyzed all prenatal specimens received for rubella serology testing at the PHOL between January 1, 2006 and December 31, 2010. In Ontario, although private and hospital laboratories have the ability to perform rubella prenatal testing, virtually all such testing is performed at PHOL at a dedicated prenatal laboratory. We extracted data from two laboratory information systems: Labyrinth (January 2006 - April 2010) and Labware (April 2010 - December 2010) and merged specimen-based data extracts from both information systems. If more than one test was performed on a single specimen, we used the result from the last test performed on the specimen.

For diagnostic samples, serologic testing for rubella antibodies, both immunoglobulin G (IgG) and IgM, were performed using the Enzygnost Rubella IgG and IgM enzyme immunoassays (EIA) on a BEP 2000 Analyzer (Siemens AG, Germany) until March 2010. Thereafter, the Euroimmun Anti-Rubella IgG and IgM enzyme-linked immunosorbent assays (ELISA) were used on a Euroimmun Analyzer I (Euroimmun AG, Luebeck, Germany). Serum samples for prenatal screening of rubella IgG antibodies were performed using the Abbott Microparticle-Enzyme Immunoassay (MEIA) on an Axsym (Abbott Diagnostics, Illinois, USA). All assays were performed according to the respective manufacturers’ instructions. Immune status was determined using the following cut-off values: < 5.0 IU/mL (Susceptible), 5.0-9.9 IU/mL (Indeterminate), > = 10.0 IU/mL (Immune).

We created a prenatal patient-based dataset by selecting all tests where the reason for testing was specified as prenatal, the patient was female and was between 15 and 49 years of age. Multiple tests were assigned to the same patient on the basis of health card number (HCN), name and date of birth (DOB). First, records with a valid HCN were assigned a unique patient identifier and grouped accordingly; next, records that did not have a valid HCN but whose name and DOB matched that of the patients identified in the first round were considered to belong to the same patient; third, among the remaining unlinked records, multiple records were assigned to the same patient on the basis of name and DOB only; lastly, all remaining records that could not be linked to previously identified patients were treated as distinct patients.

To eliminate multiple tests in the same patient that were performed within a short time period and were likely related to the same pregnancy, test results were excluded if they were performed within nine months of another test. Among patients who had multiple tests, different results were summarized into broad categories using the following approach: ‘Susceptible’, ‘Indeterminate’ and ‘Immune’ status was assigned to patients for whom all tests indicated a status of susceptible, indeterminate and immune status, respectively; ‘Immune-Susceptible’ status was assigned to patients for whom the initial and possibly subsequent tests indicated immunity followed by and ending with test(s) that indicated susceptibility; ‘Susceptible-Immune’ status was assigned to patients for whom the initial and possibly subsequent tests indicated susceptibility followed by and ending with test(s) that indicated immunity; ‘Other’ status was assigned to patients who had all other combinations of test results (e.g. Immune- Susceptible-Immune-Indeterminate).

Variables that were available for analysis were limited to information captured on the laboratory requisition forms, and comprised of the patient’s date of birth, sex and city of residence or postal code. The date of birth and date on which the specimen was received at PHOL were used to determine the age of the patient at the time that the test was performed. Patient residence was determined using a combination of the reported city of residence (prior to April 2010) and postal code (after April 2010). In the event this information was unavailable, the postal code of the requesting health care provider was used instead. In Ontario, local health promotion and disease prevention programs are delivered by 36 public health units which vary in size and demographic profile. The corresponding health unit was determined using the public health unit locator resource available through the Ontario Ministry of Health and Long-Term Care (when city was reported) and the 2009 Postal Code Conversion File maintained by Statistics Canada (when postal code was reported). Incidence rates were calculated using population data from Statistics Canada. Annualized rates were derived by dividing the numerator of interest by the combined female population between 2006 and 2010.

SAS version 9.2 was used to compile and manipulate all datasets. Descriptive analyses and statistical tests of comparisons were also conducted within SAS. Statistical tests to compare rates between age groups and health regions were based on the binomial distribution. Comparisons over time were based on the Cochran-Armitage test to test for trends over time. Statistical significance was declared at p < 0.05.

## Results

**Figure 1:**
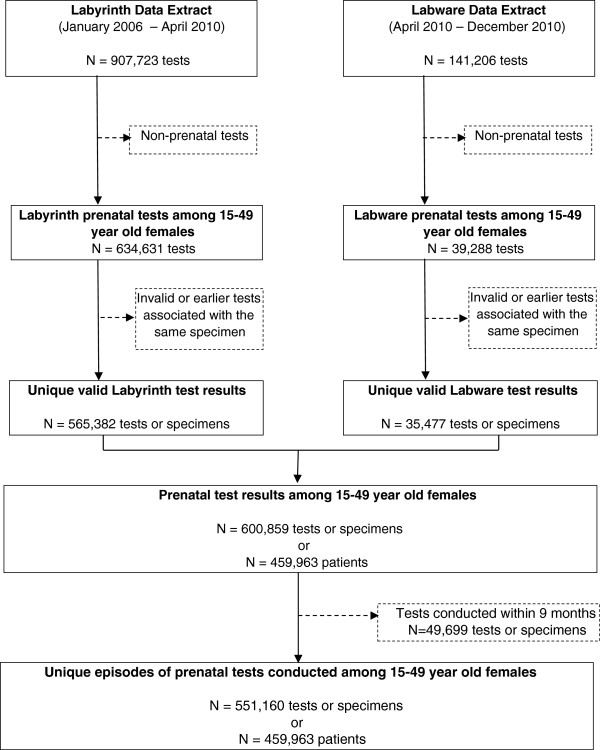
**Flowchart of data.** Flowchart depicts the process by which patient-based and test-based prenatal rubella serology datasets were derived for analysis in this study. The original number of rubella serology tests extracted from the two laboratory information systems used by the Public Health Ontario Laboratory over the study period between 2006 and 2010 is shown (N = 1,048,929). Restricting this dataset to valid prenatal tests conducted on women 15–49 year olds yielded 600,859 tests among 459,963 patients. Further restricting the dataset to tests conducted at least 9 months apart resulted in a total of 551,160 test results available for analysis.

As depicted in Figure 1, we extracted 1,048,929 test results relating to rubella serology from the laboratory information systems at PHOL between 2006 and 2010. Of these, 600,859 prenatal tests were conducted among 459,963 female patients between 15 and 49 years of age. The elimination of tests conducted less than nine months apart for the same patient resulted in 551,160 unique prenatal testing episodes for analysis. Within this cohort, 81.6% of women (N = 375,219) had one unique prenatal test during the 5-year study period, while 17.1% (N = 78,517) had two and 1.4% (N = 6,227) had three or more prenatal tests at least nine months apart.

**Figure 2:**
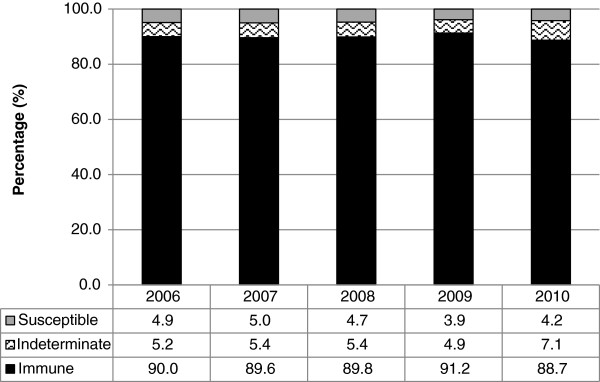
**Prenatal specimen-based rubella serology results among 15–49 year old females in Ontario, 2006–2010 (N = 551,160 tests).** This figure shows the proportion of prenatal specimens that were determined to be susceptible, indeterminate or immune to rubella among 15–49 year old females in Ontario, by year from 2006 to 2010. Immunity remained relatively stable at approximately 90% throughout the study period. There was more variation among susceptibility, ranging between 5.0% in 2007 and 3.9% in 2009.

**Figure 3:**
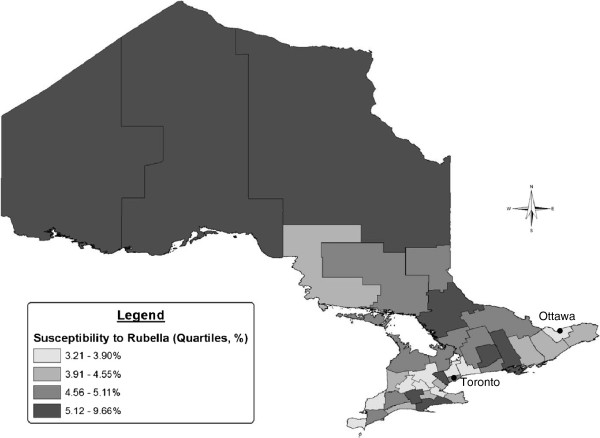
**Rubella susceptibility among 15–49 year old females in Ontario by health unit, 2006–2010 (N = 455,745 patients, excluding 4,218 patients for whom health unit could not be determined).** The geographic distribution of Ontario women who remained susceptible to rubella throughout the study period between 2006 and 2010 is presented in this figure. The health unit reflects the patient’s place of residence where available, otherwise the health unit of the health care provider who submitted the request for the prenatal test is shown. The numerator includes women who had a single test that demonstrated susceptibility, as well as women who had multiple tests that all demonstrated susceptibility. Health units were categorised according to quartiles of susceptibility. In general, women residing in the northern health units had the highest levels of susceptibility.

Over the study period, approximately 90% of the prenatal specimens demonstrated immunity to rubella, with little variation over time (Figure 2). There was more variation among susceptibility, ranging from 5.0% in 2007 to 3.9% in 2009. Across the province, susceptibility was highest among pregnant women in the northern health units during the study period (Figure 3). The proportion of women who developed immunity in subsequent pregnancies after initially being susceptible to rubella was also highest in the northern health region (1.0%), while Toronto was associated with the lowest proportion (0.4%).

Between 2006 and 2010, the overall proportion of women who were susceptible to rubella (even through multiple tests) was 4.4% (N = 20,056), while the proportion of women who were immune was 89.7% (N = 412,431). Included in this cohort were women with multiple tests and differing results: 0.03% (N = 145) of all women in this cohort were initially found to be immune and then became susceptible to rubella, while 0.6% (N = 2,727) of women were initially found to be susceptible and subsequently developed immunity. The proportion of women whose immune status was classified as indeterminate was 4.8% (N = 21,876), while 0.6% (N = 2,728) had some other combination of test results.

The distribution of tests by patient age over the study period is presented in Table 1. The highest proportion of tests was performed among women 30–34 (31.4% or mean annualized rate of 78.4 per 1,000) and 25–29 years old (29.5% or mean annualized rate of 74.2 per 1,000). In comparison, 28.1% and 33.9% of live births in Ontario in 2009 were born to women 25–29 and 30–34 years old, respectively (14). The proportion of prenatal tests performed was lowest among women 15–19 years old and women over 40 years old.

**Table 1:** Distribution of prenatal tests among 15–49 year old females in Ontario, by age at which the tests were performed, 2006–2010

**Age group (years)**	**Number of prenatal tests, 2006-2010**	**Proportion of all prenatal tests (%)**	**Mean annualized rate per 1,000**
15-19	27,247	4.9	12.7
20-24	84,727	15.4	38.5
25-29	162,377	29.5	74.2
30-34	172,819	31.4	78.4
35-39	85,311	15.5	36.7
40-44	17,553	3.2	6.8
45-49	1,125	0.2	0.4

(N = 551,159 tests, excluding 1 test for which age could not be determined).

**Figure 4:**
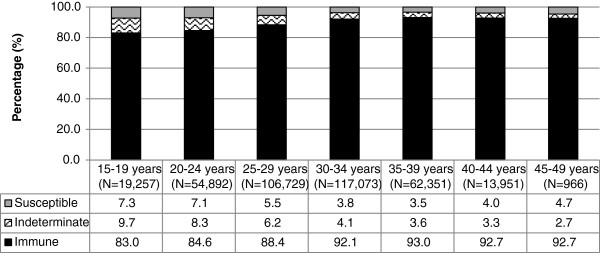
**Rubella serology results among 15–49 year old females in Ontario who had a single prenatal test by age, 2006–2010 (N = 375,219 patients).** This figure presents rubella serology results among women who had a single prenatal test between 2006 and 2010 in Ontario, by age at the time the test was conducted. In general, susceptibility to rubella declined with increasing age; women between 15 and 19 years of age had the highest susceptibility to rubella followed by women 20–24 years old.

Among the 81.6% of women who had a single prenatal test, susceptibility to rubella declined with increasing age (Figure 4, p < 0.0001); women between 15 and 19 years of age had the highest susceptibility to rubella (7.3%) followed by women 20–24 years old (7.1%). Among women who had multiple prenatal testing episodes, Table 2 presents serology results by the age of the woman at the first test. Increased age was associated with decreased susceptibility and increased immunity (p < 0.0001 for both). Specifically, younger women had the highest susceptibility (4.2% among 15–19 year-olds) and lowest immunity (87.6% among 15–19 year olds). Notably, women were significantly less likely to develop immunity if previously susceptible, with increasing age (p < 0.0001).

**Table 2:** Proportion of 15–49 year old females in Ontario who had multiple rubella serology tests, by age at first test and selected sequence of test results, 2006–2010

**Age at first test**	**Sequence of test results**				
	**Susceptible**	**Immune-susceptible**	**Indeterminate**	**Susceptible-immune**	**Immune**
15-19 years (N = 5,663)	4.2	0.3	3.1	4.9	87.6
20-24 years (N = 14,239)	3.1	0.3	2.4	4.5	89.8
25-29 years (N = 26,596)	2.0	0.2	1.6	3.7	92.5
30-34 years (N = 22,326)	1.4	0.2	1.0	2.3	95.2
35-39 years (N = 7,406)	1.8	0.1	1.0	1.8	95.3
40-49 years (N = 855)	1.3	0.4	1.6	1.1	95.5

(N = 82,016 patients, excluding 2,728 patients for whom a different sequence of test results was observed).

**Figure 5:**
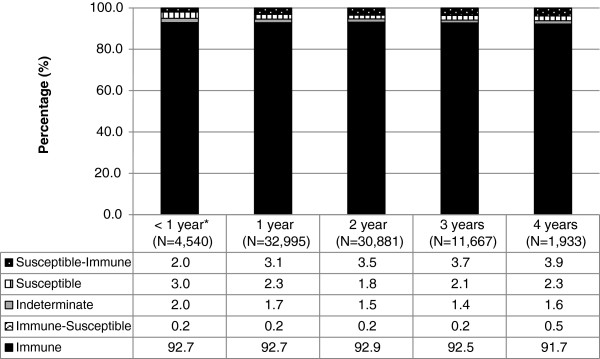
**Patient-based rubella serology results among 15–49 year old females in Ontario with multiple tests by interval between first and last test (N = 82,016 patients, excluding 2,728 patients for whom a different sequence of test results was observed).** Among women who had multiple prenatal tests between 2006 and 2010, this figure shows the distribution of women classified according to the sequence of test results, by the number of years between the first and last test. The proportion of women who remained susceptible throughout repeated prenatal testing declined as the number of years between tests increased; conversely, the proportion of women who were initially susceptible and then became immune increased with longer intervals. * Interval refers to 9 months to, 1 year, as tests conducted within 9 months were considered to be associated with the same pregnancy.

The effect of the interval between tests among women who had multiple prenatal tests is shown in Figure 5. Although not shown, these trends were similar across all age groups. The proportion of women who remained susceptible throughout repeated prenatal testing declined as the number of years between tests increased (p < .0001). Also, while the proportion of women who remained immune throughout repeated prenatal testing was unchanged at approximately 93%, the proportion of women who were initially susceptible and then became immune increased from 2.0% to 3.9% (p < .0001) as the interval between the first and last tests increased.

## Discussion

Our analyses showed that overall rubella susceptibility among prenatal women in Ontario between 2006 and 2010 was 4.4% and decreased from 4.9% to 4.2% over the same time period. This is lower than the corresponding level of susceptibility (4.9%) associated with the national target of 97% vaccine coverage, assuming 98% vaccine effectiveness of one dose of rubella-containing vaccine. However it exceeds the national target of less than 4% susceptibility among first-time pregnant women, particularly at the health unit level where proportions as high as 9.7% were observed.

Drawing comparisons with other jurisdictions is challenging due to methodological differences, such as different study populations and unknown or varied thresholds used to define immune status. Rubella susceptibility demonstrated in this analysis is lower than what was reported in previous studies conducted among pregnant women in Quebec (1993–1994) and Toronto, Ontario (2002–2007), with estimates between 7.0% and 8.4% [13,15]. A study conducted in Alberta in 2002–2005 also reported higher rubella susceptibility (8.8%) but a higher threshold (<10 U/mL) was used to define susceptibility [14]. The American population-based National Health and Nutrition Examination Survey (NHANES) estimates that 8.5% of women 20 to 49 years of age are susceptible to rubella infection above a modeled threshold of rubella elimination of greater than 87.5% population immunity [16]. The immunity demonstrated in our study is greater than this threshold, though it is important to note that the threshold was not limited to prenatal women and was specific to the United States rather than the Canadian or Ontario population.

The significant increase in immunity with increasing age may be attributable to increased past exposure to natural infection, as well as more opportunities to immunize older women during their childbearing years either as a result of pre-conception screening or in the post-partum period. This is further supported by the increasing proportion of women who were initially susceptible becoming immune over time since their first prenatal test. Many hospitals have adopted standing orders for rubella non-immune women and the benefits of post-partum standing orders has been shown to be effective in increasing rubella immunization among non-immune women prior to hospital discharge [17]. As shown in Table 2 and Figure 4, higher susceptibility was observed among adolescents and young women (15–29 year olds). This could reflect waning immunity from childhood vaccinations, as this cohort would have been eligible for one dose of rubella-containing vaccine in childhood, and antibody levels have been shown to decline within a few years of vaccination [18,19]. The lack of natural boosting due to an absence of circulating virus, may also result in higher susceptibility particularly among younger women [6]. Further, younger pregnant women may access healthcare differently than older pregnant women and thus may have different pre-conception/prenatal health behaviours [20,21]. Although rubella is not endemic in Canada, importation of cases occurs, therefore these women remain at risk for rubella infection during pregnancy which may result in congenital rubella syndrome among their offspring.

This analysis also found that younger women and women living in northern regions of Ontario were more likely to be susceptible to rubella. This is consistent with a similar population-based analysis of prenatal specimens conducted in Alberta where younger women and women from northern Alberta were also significantly more likely to have seronegative specimens [14]. Although school-based immunization coverage data among 17-year old students within the province reveals high vaccine coverage (96.6%) [6], the general population may not be representative of high risk populations such as pregnant adolescents, and immunization status was not available for analysis in this study. Increased susceptibility in northern regions of Ontario could reflect barriers to immunization due to geographic isolation and sub-optimal access to primary health care services particularly among First Nations communities [22-24]. Health units in the northern region are more rural and tend to be more sparsely populated compared to the southern and central regions of the province. For example, Toronto and the surrounding central health units comprise approximately 70% of the provincial population, whereas just 6% of Ontarians reside in the north.

During the 5-year study period, 81.6%, 17.1%, and 1.4% of women had one, two and three or more prenatal tests at least nine months apart, respectively. According to national data from Statistics Canada, 44%, 35% and 21% of all live births represented first, second and third or higher order births [25]. This difference may be explained by the shorter observation period used in this study, relative to the greater number of years in which women bear children. In our study, we observed 0.6% of women who converted from susceptible to immune, which may be attributed to postpartum immunization. This is consistent with recommendations for universal rubella screening of all pregnant women and post-partum immunization of non-immune women [9]. What is more concerning is the 0.03% of women who were identified as initially being immune but subsequently became susceptible. While this may suggest waning immunity, this may also represent women whose test results were borderline reactive or who were incorrectly identified as the same patient during the matching process.

There are several limitations associated with this study. Serological results do not distinguish between vaccine- and disease-induced immunity. However as rubella is not an endemic disease in Canada and the number of cases in Ontario is low, our results likely reflect vaccine-induced immunity. We were also limited by specimens that were submitted to PHOL for screening. As such, we may have missed prenatal specimens that were submitted by private and hospital laboratories, and our analysis was limited by the information was captured on the requisition form. However since prenatal testing for other diseases (i.e. hepatitis, HIV and syphilis) are performed at PHOL and are routinely requested with rubella screening, private and hospital laboratories tend to forward rubella requisitions to PHOL. Thus, the volume of missed tests was likely minimal. While the objectives of this study were met, the lack of variables available for analysis limited our ability to interpret observed trends. For example, we were not able to determine if women who seroconverted had received vaccination post-partum. Future studies may consider linkages with additional data sources. We also identified some inconsistencies in the use of prenatal requisition forms, such as the use of these forms among males and females outside of the 15 to 49 year age group. However as our analyses were strictly limited to prenatal tests conducted among 15–49 year old females, it is unlikely that non-prenatal tests were included. Lastly, although data were compiled from two different laboratory information systems (Labyrinth and Labware) during the study period, the same threshold values were used to identify susceptible, indeterminate and immune patients, thus eliminating inconsistencies in the interpretation of test results.

## Conclusions

Our study demonstrates that rubella susceptibility among prenatal women in Ontario is within the range, if not lower, than what has been reported in other jurisdictions in North America, and supports elimination goals as population immunity in this group is relatively high. Despite this, higher susceptibility among young women and women living in the north highlights an opportunity for greater focus on identification and immunization of susceptible women in these groups.

## Competing interests

The authors declare that they have no competing interests.

## Authors’ contributions

GHL, TH, SLD contributed towards the write-up of the manuscript; GHL prepared the data for analysis and performed all statistical analysis; SLD, NSC, SD conceived of the study; TM, TK provided guidance in changes to laboratory techniques and test results over time; NSC, SLD contributed towards the analytic approach; SLD provided overall guidance throughout the course of the study and facilitated data access needs. All authors participated in the interpretation of results, and reviewed and approved the final manuscript.

## Pre-publication history

The pre-publication history for this paper can be accessed here:

http://www.biomedcentral.com/1471-2334/13/362/prepub
